# Changes in the proteome of *Apis mellifera* acutely exposed to sublethal dosage of glyphosate and imidacloprid

**DOI:** 10.1007/s11356-024-34185-x

**Published:** 2024-07-09

**Authors:** Carlos Andrés Maya-Aguirre, Angela Torres, Luz Dary Gutiérrez-Castañeda, Luz Mary Salazar, Yael Abreu-Villaça, Alex Christian Manhães, Nelson Enrique Arenas

**Affiliations:** 1 Instituto de Biotecnología, Facultad de Ciencias, Universidad Nacional de Colombia, Ciudad Universitaria, Avenida Carrera 30 N° 45–03, Bogota, D.C Colombia; 2 Grupo Ciencias Básicas en Salud–CBS-FUCS, Fundación Universitaria de Ciencias de La Salud, Hospital Infanti L Universitario de San José, Carrera 54 No.67A–80, Bogota, D.C. Colombia; 3 Departmento de Química, Facultad de Ciencias, Universidad Nacional de Colombia, Ciudad Universitaria, Avenida Carrera 30 N° 45–03, Bogota, D.C. Colombia; 4 Laboratório de Neurofisiologia, Departamento de Ciências Fisiológicas, Instituto de Biologia Roberto Alcantara Gomes, Universidade Do Estado Do Rio de Janeiro (UERJ), Rio de Janeiro, RJ 20550-170 Brazil; 5 Facultad de Medicina, Universidad de Cartagena, Campus Zaragocilla, Barrio Zaragocilla, Carrera 50a #24-63, Cartagena de Indias, Bolivar Colombia

**Keywords:** *Apis mellifera*, Proteomics, Glyphosate, Imidacloprid, Pesticides, Protein–protein interaction networks, Metabolism, Neurological effects

## Abstract

Uncontrolled use of pesticides has caused a dramatic reduction in the number of pollinators, including bees. Studies on the effects of pesticides on bees have reported effects on both metabolic and neurological levels under chronic exposure. In this study, variations in the differential expression of head and thorax–abdomen proteins in Africanized *A. mellifera* bees treated acutely with sublethal doses of glyphosate and imidacloprid were studied using a proteomic approach. A total of 92 proteins were detected, 49 of which were differentially expressed compared to those in the control group (47 downregulated and 2 upregulated). Protein interaction networks with differential protein expression ratios suggested that acute exposure of *A. mellifera* to sublethal doses of glyphosate could cause head damage, which is mainly associated with behavior and metabolism. Simultaneously, imidacloprid can cause damage associated with metabolism as well as, neuronal damage, cellular stress, and impairment of the detoxification system. Regarding the thorax–abdomen fractions, glyphosate could lead to cytoskeleton reorganization and a reduction in defense mechanisms, whereas imidacloprid could affect the coordination and impairment of the oxidative stress response.

**Supplementary Information:**

The online version contains supplementary material available at 10.1007/s11356-024-34185-x.

## Introduction

Global population growth requires a high food demand, leading to extensive agriculture that must implement strategies to increase food production. Recent reductions in crop yield are associated with multiple factors, including climate change, water scarcity, resource pollution, soil nutrients, soil-borne pathogens, and weeds that may harbor pests or phytopathogens (Cerda et al. 2017; Chauhan 2020). Therefore, pest control strategies in agriculture rely on the use of herbicides and insecticides. However, the extensive use of these products to combat weeds or insects has led to a reduction in bee populations, which are considered the major pollinators of a wide variety of wild plants and monocultures, making them crucial for various agricultural activities (Kline and Joshi 2020).

Pollinators contribute between 235 and 577 billion dollars to annual crop production (Aizen et al. 2009). In the USA, it represents approximately $16 billion in annual crop production is generated by pollinators, and bees are responsible for $12 billion annually (Khalifa et al. 2021). The use of insecticides or herbicides causes a reduction in pollinators, resulting in crop losses ranging between 5 and 8%. Additionally, it generates ecological imbalances (Aizen et al. 2009).

Imidacloprid (IMI) is a common insecticide used in agriculture that indirectly targets bees and other beneficial insects (Pang et al. 2020). IMI is a systemic neonicotinoid insecticide that competitively inhibits nicotinic acetylcholine receptors in insects (Buckingham et al. 1997). This insecticide is used to protect crops against insects such as termites and larvae (Ensley 2007). Under IMI, chronic exposure of *A. mellifera* bees to sublethal concentrations of IMI resulted in a reduction in the total protein content compared to that in the control group, affecting proteolytic activity (Wilde et al. 2016). Furthermore, chronic topical exposure of drones and worker bees of *A. mellifera* to sublethal concentrations of IMI led to the differential expression of proteins in the hemolymph associated with the stress response (McAfee et al. 2022).

On the other hand, glyphosate (GLY) primarily affects plant species, and various studies have indicated its impact on insects, including bees (Battisti et al. 2021; Luo et al. 2021). GLY is a nonselective herbicide that is applied postemergence and targets more than 150 weed species (Kanissery et al. 2019). Specifically, this compound inhibits the 5-enolpyruvylshikimate-3-phosphate synthase, which is involved in the synthesis of chorismate, a precursor of tryptophan, phenylalanine, tyrosine, and vitamins such as folic acid and K2 (Hertel et al. 2021). Honeybees of the species *A. cerana cerana* and *A. mellifera ligustica* chronically exposed to sublethal doses of commercial GLY exhibited differential expression of genes associated with carbohydrate metabolic pathways, the digestive system, lipid metabolism, the immune system, nervous system, and membrane transport, among other key processes (Zhao et al. 2020). Consistently, chronic exposure to GLY in *A. mellifera* affects mitochondrial morphology in the hypopharyngeal glands, resulting in damage associated with apoptotic events (Faita et al. 2018). Similarly, analysis of the protein profile of royal jelly from honeybees chronically exposed to GLY at sublethal doses revealed a reduction in the expression of major royal jelly protein 3 (MRJP-3), which may be associated with nutritional imbalances that alter immunity and homeostasis (Faita et al. 2022). In addition to the metabolic aspects, neurological processes are also affected, such as the reduction in AChE activity described in bees treated with sublethal doses of GLY (Boily et al. 2013). Consequently, impairment of navigation systems and memory occurs in bees exposed to GLY (Zgurzynski and Lushington 2019).

This evidence was addressed from the perspective of the collateral damage caused by insecticides and herbicides on pollinating insects, such as bees (Martin-Culma and Arenas-Suárez 2018). Since the proteomic effects of GLY and IMI when administered acutely are unknown, we aimed to determine the variation in proteomic fractions obtained from the head and the thorax–abdomen of *A. mellifera* bees exposed to sublethal doses of GLY and IMI.

## Materials and methods

### Biological samples

The honeybees used in this research were derived from Africanized bees of the species *Apis mellifera scutellata*, which they arrived in Colombia from Brazil and were mixed with local bees in the 1970s (Tibatá et al. 2017). The experimental setup included 75 worker honeybees that were placed in conical tubes with lids and air holes and treated with GLY and IMI. Subsequently, the honeybees were immobilized with tape between the head and thorax after being placed in dissecting needles. Twenty-five honeybees were treated with a GLY solution (40 μg/bee), 25 honeybees with IMI (2.4 ng/bee), and 25 honeybees were used as a control group and were administered 30% w/v sucrose solution. The dosages used in this study represented less than 5% of the LD_50_ reported for honeybees treated with glyphosate (1773.06 μg/bee) and imidacloprid (132 ng/bee) (Chen et al. 2022), (He et al. 2021). This criterion has been applied by various studies associated with the physiological variation of honeybees treated with these pesticides (Boily et al. 2013; Wang et al. 2022) and the doses used represent 2.2% and 1.8% of glyphosate and imidacloprid, respectively. Pesticide intake was performed using the proboscis extension reflex (PER) technique (Bhagavan and Smith 1997). After the pesticides (GLY and IMI) were administered, the honeybees were kept in darkness for 24 h, including the honeybees from the control group (treated only with sucrose). At the end of this period, all the honeybees were sacrificed by freezing with liquid nitrogen. The pesticides used were GLY in the commercial formula ammonium salt (Panzer 747 WG) and IMI in suspension (Confidor-Bayer™), which were prepared in 30% w/v sucrose solution.

### Extraction of head and thorax–abdomen proteins

Honeybees were classified into control, GLY-, and IMI-treated groups into three subgroups of five bees each. Two fractions were generated by separating the heads from the thorax–abdomen after pesticide treatment and washing with saline solution (0.9% w/v NaCl). Both the head and thorax–abdomen fractions were macerated with a pestle in PBS buffer (2.6 mM NaH_2_PO_4_; 32.5 M Na_2_HPO_4_, 400 mM NaCl, pH 7.6) at a 10:1 ratio dilution (w/v). Samples were lysed for 20 min by ultrasonication for 30 min on an ice bath and centrifuged at 15,000 rpm for 10 min at 4 °C. Protein samples were collected from the supernatants in PBS buffer, and the precipitates obtained were supplemented with the same buffer at a ratio of 2:1 (w/v) and centrifuged at 15,000 rpm for 10 min at 4 °C. The supernatants were lysed with RIPA buffer at a ratio of 10:1 (w/v), cooled for 10 min in an ice bath, and sonicated for 30 min at 4 °C. The samples were centrifuged at 15,000 rpm for 10 min at 4 °C, and the supernatants were transferred to tubes containing the soluble proteins extracted previously with PB buffer (Li et al. 2007). Total proteins were precipitated using cold acetone (4 × volumes), followed by incubation in an ice bath for 10 min and centrifugation at 15,000 rpm for 10 min at 4 °C to obtain a precipitated protein pellet. Each pellet obtained was washed with 500 μL of cold acetone, vortexed, and centrifuged at 15,000 rpm for 10 min at 4 °C; this process was repeated four times. The pellet of each sample was reconstituted in ammonium bicarbonate buffer (50 mM, pH 8.5) at a 4:1 ratio (v/v). Electrophoretic profiles were evaluated using SDS-PAGE according to the protocol suggested by Ramon–Sierra and colleagues (2015).

### Preparation of protein extracts and digestion of samples

Total protein content was quantified using the Qubit assay. Proteomic analysis was performed using 100 μg of total protein previously processed for disulfide bridge reduction by incubation with 10 mM dithiothreitol (DTT) at 56 °C for 30 min. The samples were alkylated in the dark at room temperature for 1 h using 10 mM iodoacetamide (Kolsrud et al. 2012; Suttapitugsakul et al. 2017). The treated protein extracts were digested with trypsin (sequencing grade, Promega) at an enzyme ratio of 1:100 (w/w). Digestion was performed by incubation with protease for 24 h at 37 °C. After digestion, the samples were lyophilized and desalted using C18 spin columns. After desalting, the samples were subjected to detergent removal from the extraction, washed twice with ethyl acetate, and dried using a SpeedVac system.

### Sample analysis using the LC–MS/MS method

The samples were reconstituted in 30 μL of 3% v/v acetonitrile in LC–MS-grade ultrapure water (water mixed with 0.1% v/v formic acid). The samples were processed on an Ultra-Performance Liquid Chromatography-nano-UPLC system (Waters, Milford, MA) coupled in-line with a SYNAPT G2-Si mass spectrometer (Waters, Milford, MA). The UPLC system was equipped with a Symmetry C18 nanoAcquity reverse-phase column (180 μm × 20 mm, 5 μm Thermo Scientific ™) and a Waters HSS-T3 C18 (75 μm × 150 mm, 1.8 μm Waters ™) reverse-phase analytical column. Approximately 1 μg of each sample was injected at a rate of 500 nL/min with an elution gradient of 3 to 25% of mobile phase B (0.1% v/v formic acid in 98% v/v acetonitrile) in mobile phase A (0.1% v/v formic acid) over a 90-min program, followed by an increase in mobile phase B from 25 to 85% for 7 min, a wash phase for 3 min with mobile phase B at 85%, and finally an equilibration phase with 3% mobile phase B for 10 min.

### MS/MS data acquisition and processing

Mass spectra were acquired on a SYNAPT G2-Si mass spectrometer, and data processing was performed using Waters MassLynx software (version 4.1, SCN 851). The mass spectrometer was operated in resolution mode with a typical resolving power of up to 20,000 FWHW at a mass-to-charge ratio (m/z) of 785.8427 (doubly charged Glu-fibrinopeptide B ions were used as calibration standards at a concentration of 300 fmol/μL prepared in an aqueous solution with 30% v/v acetonitrile and 0.1% v/v formic acid). All analyses were performed in positive electrospray ionization mode using a NanoLockSpray source. The instrument was configured for an acquisition range of 50–1800 m/z with a capillary voltage of 2.8 kV, source temperature of 100 °C, and cone voltage of 30 V. The radio frequency of the quadrupole analyzer was adjusted for efficient ion transmission from 300 to 2000 m/z. The data were analyzed using the Progenesis QI platform (version 4.2), and the proteomic expression profiles were compared. The mass tolerance for low-energy precursor ions was set to 20 ppm, whereas the mass tolerance for high-energy fragment ions was set to 50 ppm. Carbamidomethylation of cysteines and trypsin digestion with cleavage were also considered. The search threshold criteria included a minimum of 3 fragment ion matches per peptide, 7 per protein, and at least 1 peptide per protein, with a maximum of 5 false positives per protein identification. Protein identification was performed using the UniProt database (https://www.uniprot.org/) of the *A. mellifera* proteome (UniProtKB 25,386 entries). Proteins were considered downregulated at a ratio of < 0.5, whereas those upregulated had a ratio of > 2 and a statistical significance of *p* < 0.05.

### Bioinformatic interaction analysis and protein clustering

Functional enrichment analysis was conducted using UniProt and PANTHER databases (http://pantherdb.org/about.jsp). Both tools were used to ensure the comprehensive coverage of gene ontology (GO) annotations. Subsequently, protein–protein interaction networks were constructed using the STRING tool (https://string-db.org/) and normalized using the Database for Annotation, Visualization, and Integrated Discovery (DAVID) tool. The networks were generated with a confidence interval parameter of 0.7 and a maximum number of interactor parameters of 50. Visualization of the interaction networks obtained from STRING was performed using Cytoscape version 3.8.0 (https://cytoscape.org/) (Shannon et al. 2003). Protein clustering was performed using the MCODE plugin implemented in Cytoscape to classify the protein groups within the network according to the biological processes per cluster (Wang et al. 2010). The parameters used for clustering were as follows: minimum number of connections (degree cutoff), 2, node density limit (node density cutoff); 0.1, node score limit (node score cutoff); 0.2, node connection index (K-Core); 2, and maximum depth (max depth), 100.

### Statistical analysis

Statistical analyses were conducted using the SPSS statistical package. The results obtained from the GLY or IMI treatments were compared using Student’s *t*-test and one-way and two-way analysis of variance (ANOVA), followed by multiple comparison testing using Tukey’s test. Proteomic changes were considered significant if the differences were *p < 0.05, **p < 0.001.

## Results

### Global changes in the proteome of *A. mellifera* induced by GLY and IMI treatments

**Fig. 1:**
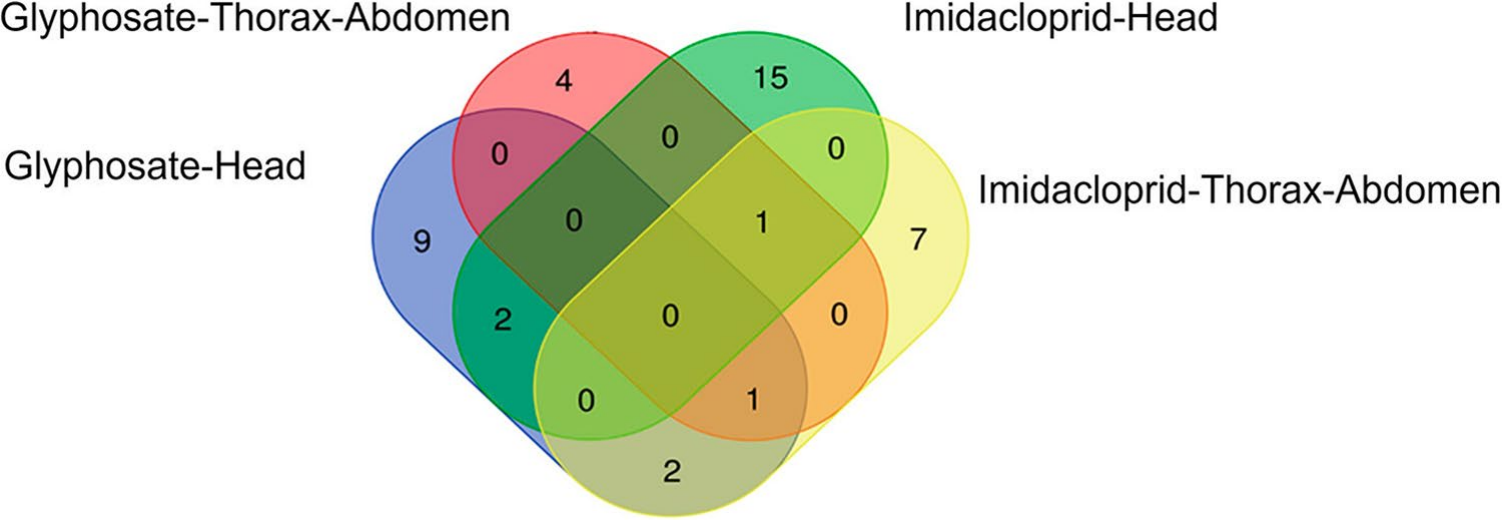
Venn diagram comparing commonly detected proteins between IMI and GLY treatments in *A. mellifera*

Although the mechanisms of action of GLY and IMI differ, global physiological processes were affected. Moreover, among the differentially detected proteins compared to those in the control group, some were found to be common between the treatments (Fig. 1). In the heads of honeybees treated with GLY, the protein MRJP-1 was detected, and this same protein was also detected in both body sections (head and thorax–abdomen) from honeybees treated with IMI; the treatment with IMI caused differential expression of myosin heavy chain protein in both body sections; in the head, two proteins were detected in common in honeybees treated with GLY and IMI (mitochondrial dicarboxylate carrier isoform X1 and Phosphorylase b kinase regulatory subunit); in thorax–abdomen from honeybees treated with GLY and in the whole body from honeybees treated with IMI, the myosin heavy chain, non-muscle isoform X4 protein was detected; and in the head from honeybees treated with GLY and in the thorax–abdomen from honeybees treated with IMI, the fatty acid synthase protein was detected. The mentioned proteins and their associated processes are described below:

- GLY (thorax–abdomen) and IMI (whole bee): major royal jelly protein 1 MRJP-1, associated with nutrition and defense against bacterial agents.
- GLY and IMI (head): mitochondrial dicarboxylate carrier isoform X1 (SLC25A10) and phosphorylase b kinase regulatory subunit (PHKB) are associated with carbohydrate metabolism.
- GLY (thorax–abdomen) and IMI (whole bee): myosin heavy chain, non-muscle isoform X4 (MYH9), associated with the reorganization of the cytoskeleton and muscle fibers.
- GLY (head) and IMI (thorax–abdomen) fatty acid synthase (FASN) and mevalonate kinase (MVK) are associated with fatty acid metabolism.

### Changes in proteomic expression in *A. mellifera* head fractions

A total of 92 protein datasets were identified in the head and thorax–abdomen fractions of bees treated with GLY and IMI. After determining which of these proteins showed differential expression compared to the control, 14 proteins were detected in the head and six in the thorax–abdomen fractions in *A. mellifera* under GLY treatment. Additionally, 18 and 11 proteins were detected in the head and thorax–abdomen, respectively, after IMI treatment. Proteins detected in the head samples represented 52 of the 92 proteins detected in this study. After GLY treatment, 23 proteins were detected in the head fractions, and 29 were detected in those treated with IMI. In terms of differential expression, 14 proteins were downregulated in the heads of bees treated with GLY (Table 1) and 18 were downregulated in the heads of bees treated with IMI (Table 2).

**Table 1:** Differentially expressed proteins in the heads of honeybees treated with GLY. *FC* fold change (*p*-value < 0.05)

UNIPROT	Gene name	Protein name	FC	*p*-value
A0A7M7R941	LOC552286	Acetyl-CoA carboxylase (ACC) (EC 6.4.1.2)	− 1.45	2.83E-03
Q25BT6	HBG3	Alpha-glucosidase (GAA) (EC 3.2.1.20)	− 1.85	2.34E-02
A0A7M7TFF0	LOC411959	Fatty acid synthase (FASN)	− 2.63	2.85E-03
A0A7M7LR86	725055	Gamma-glutamylcyclotransferase (GGCT) (EC 4.3.2.9)	− 2.86	4.83E-03
O18330	MRJP1	Major royal jelly protein 1 (MRJP1)	− 1.83	4.10E-04
A0A7M7SRV2	LOC412503	Mitochondrial dicarboxylate carrier isoform X1 (SLC25A10)	− 2.04	4.84E-03
A0A7M7IK12	552163	Mevalonate kinase (MVK) (EC 2.7.1.36)	− 1.46	3.96E-02
Q1W633	OBP21	Odorant binding protein 21 precursor (OBP21)	− 1.65	1.73E-03
Q9NDF3	PER	Period circadian protein (Period clock protein) (PER)	− 1.43	1.38E-02
A0A7M7GMB2	LOC550706	Phosphorylase b kinase regulatory subunit (PHKB)	− 2.43	2.85E-03
A0A7M7FYH3	725018	Phosphomevalonate kinase (EC 2.7.4.2) (PMVK)	− 2.64	4.58E-03
A0A7M7MSF1	LOC551913	Protein CREG1 (CREG1)	− 1.56	1.29E-02
A0A7M7SPD7	LOC107964791	Titin-like (TTN)	− 1.98	5.94E-04
A0A7M7GMS5	LOC726673	28S ribosomal protein S27. mitochondrial (MRPS27)	− 2.21	1.74E-03

**Table 2:** Differentially expressed proteins in the heads of honeybees treated with IMI. *FC* fold change (*p* < 0.05)

UNIPROT	Gene name	Protein name	FC	*p*-value
A0A7M7R4W5	LOC408875	Alpha-crystallin B chain isoform X1 (CRYAB)	− 1.05	3.39E-03
A0A7M7R6C3	107964020	Alpha-tocopherol transfer protein isoform X1 (TTPA)	− 1.09	2.63E-03
A0A7M7SQ18	LOC408961	Apolipophorin (APO)	− 1.86	5.95E-04
A0A7M7GV04	LOC408982	Calcineurin B homologous protein 1 (CHP1)	− 3.05	2.84E-03
A0A7M7H1C3	725804	Cuticle protein 18.7 (CPR18.7)	− 1.56	3.55E-03
A0A7M7GSP5	LOC100577777	Cysteine-rich venom protein 6 isoform X2 (CRV6)	− 1.04	5.68E-04
A0A7M7FZ75	726860	Cytochrome b5 (CYB5)	− 2.41	7.36E-03
A0A7M7RC74	551607	DNA-directed RNA polymerase II subunit RPB11 (POLR2J)	− 2.34	2.83E-02
A0A7M7R7J8	412349	ER membrane protein complex subunit 4 (EMC4)	− 1.63	7.27E-03
A0A7M7MMW0	LOC724750	ER membrane protein complex subunit 10 isoform X2 (EMC10)	− 1.13	1.39E-03
A0A7M7IKE1	LOC410171	Filamin A (FLNA)	− 3.06	7.83E-03
A0A7M7GZK8	LOC411700	Heat shock protein 83 (HSP83)	− 2.69	1.52E-04
A0A7M7GYL8	100577981	Insulin-like growth factor-binding protein complex acid labile subunit (IGFALS)	− 1.84	5.60E-04
A0A7M7SRV2	LOC412503	Mitochondrial dicarboxylate carrier isoform X1 (SLC25A10)	− 1.48	6.27E-03
A0A7M7H2S3	412092	Myosin heavy chain. non-muscle isoform X4 (MYH9)	− 3.45	2.94E-02
A0A7M7GMB2	LOC550706	Phosphorylase b kinase regulatory subunit (PHKB)	− 1.97	7.69E-04
A0A8U1DHQ6	LOC413995	Sensory neuron membrane protein 1 isoform X2 (SNMP1)	− 2.89	5.73E-03
A0A7M7IG02	725997	UDP-glucuronosyltransferase 2C1 (UGT2C1)	− 2.92	5.02E-03

All proteins were downregulated. The proteomic changes in the heads of honeybees treated with GLY were between − 1.45 and − 2.86, highlighting proteins such as gamma-glutamylcyclotransferase (GGCT) and other related metabolic enzymes (Table 1). On the other hand, the proteins whose expression changed significantly in the heads of honeybees treated with IMI ranged between − 1.05 and − 3.45, especially proteins such as the myosin heavy chain, the non-muscle isoform X4 (MYH9), and other structural proteins (Table 2).

Functional enrichment performed using the UniProt and Panther tools revelated these proteins according to their cellular location (Tables S1 and S2, such as the extracellular matrix, plasma membrane, extracellular matrix, cytosol, nucleus, and mitochondria (these last three compartments are commonly associated with the treatment of both pesticides). Regarding molecular functions, most proteins were associated with ATP binding, protein binding, DNA binding, and filaments. Interestingly, with both treatments, calcium binding and ion transport at the transmembrane level were found to be common molecular functions associated with both pesticides. The biological processes associated with the differentially expressed proteins included carbohydrate metabolism, fatty acid metabolism (major process), intramembrane transport processes, proteins associated with defense against bacterial agents and gene regulation, organization of the cytoskeleton, response to temperature changes, and involvement in protein folding.

### Protein networks and physiologic processes inhibited in samples from honeybee heads

The protein networks differentially expressed in the heads of bees upon treatment with GLY had 64 nodes and 398 interactions (Figure S1a), whereas those detected in bees treated with IMI showed 68 nodes and 417 interactions (Figure S1b). Clustering analyses of these networks have been shown to affect many biological functions and impaired processes associated with the metabolism of nitrogenous compounds, carbohydrates, regulation of cellular structure, motor regulation, circadian rhythm, and transcription, among other functions.

Table S3 shows the clustering results for the interaction network of differentially expressed proteins in the heads of bees treated with GLY or IMI. This table presents six types of clusters for proteins differentially expressed by GLY treatment and two by IMI treatment, with both high and low score values and FDR indices for all clusters, especially those with a greater number of nodes and edges, which suggests a high correlation between the proteins within the interaction network.

The clustering pattern indicated that the GLY treatment of *A. mellifera* mainly influenced various metabolic processes related to sugar metabolism, energy production, and locomotion, among others. On the other hand, treatment with IMI affected processes of locomotion, cellular differentiation, and some processes related to the transcription level (Table 3).

**Table 3:** Biological processes related to the clusters obtained from the protein interaction network of proteins detected in heads of honeybees treated with GLY and IMI

Glyphosate (GLY)	Imidacloprid (IMI)
Sucrose metabolism Fatty acid metabolism Purine metabolism Citrate metabolism (Krebs cycle) Isoprenoid biosynthesis. mevalonate (cholesterol. sterol synthesis) Oxidoreduction processes Pyruvate metabolism Circadian rhythm Motor regulation Locomotor rhythm Assembly of small ribosomal subunits Translation Glutathione metabolism Acetyl CoA metabolism Germ cell migration	Heat Shock Protein 90 (HSP90) DNA transcription Nitrogen metabolism Positive regulation of translation Endoplasmic reticulum protein Phosphorylation Calcium-mediated signaling Glycogen metabolism Cell differentiation-morphogenesis Cytoskeleton organization Actin and myosin filament organization tRNA transcription Promoter elongation of transcription from RNA Pol Regulation of transcription Pol II Pol III

### Proteins of the thorax–abdomen fractions of *A. mellifera* affected by treatment with GLY and IMI

The proteins detected in the thorax–abdomen samples represented 37 groups of the 92 proteins detected in this study. Regarding the individual treatments, six proteins were detected in bees treated with GLY (Table 4), and 11 proteins were detected in bees treated with IMI (Table 5). All proteins detected in the thorax–abdomen of honeybees treated with GLY were downregulated, with values between − 1.03 and − 2.89, indicating the presence of proteins such as xenotropic and polytropic retrovirus receptor 1 homolog and other proteins associated with structural processes (Table 4). Most of the proteins detected in the thorax–abdomen of honeybees treated with IMI were downregulated except for two proteins upregulated such as superoxide dismutase (SOD) and venom carboxylesterase-6 (Table 5).

**Table 4:** Proteins showing differential expression in the thorax–abdomen of honeybees treated with GLY. *FC* fold change (*p*-value < 0.05)

UNIPROT	Gene name	Protein name	FC	*p*-value
B0LUE8	A4	Apolipophorin-III-like protein (Apolipophorin-III-like protein precursor) (APOIII)	− 2.73	3.24E-03
O18330	MRJP1	Major royal jelly protein 1 (MRJP1)	− 1.93	2.85E-03
A0A7M7H2S3	412092	Myosin heavy chain. non-muscle isoform X4 (MYH9)	− 2.53	1.03E-04
A0A7M7MPC1	LOC411655	Serine/arginine repetitive matrix protein 1 (SRRM1)	− 2.05	3.40E-02
A0A7M7L431	LOC551464	TRAF3-interacting protein 1 (TRAF3IP1)	− 1.03	8.12E-03
A0A7M7R8P0	551795	Xenotropic and polytropic retrovirus receptor 1 homolog (XPR1)	− 2.89	3.79E-02

**Table 5:** Proteins showing differential expression in the thorax–abdomen of honeybees treated with IMI. *FC* fold change (*p*-value < 0.05)

UNIPROT	Gene name	Protein name	FC	*p*-value
A0A7M7GTN2	LOC410557	ATP synthase subunit d. mitochondrial (ATP5H)	− 2.71	3.13E-03
P00038	CYTC	Cytochrome c (CYCS)	− 2.26	1.34E-03
A0A7M7TFF0	LOC411959	Fatty acid synthase (FASN)	− 3.54	2.64E-04
A0A088AHC8	410122	Glyceraldehyde-3-phosphate dehydrogenase (GAPDH)	− 2.3	4.10E-03
O18330	MRJP1	Major royal jelly protein 1 (MRJP1)	− 1.83	1.29E-03
A0A7M7IK12	552163	Mevalonate kinase (MVK)	− 2.21	2.10E-02
A0A7M7H2S3	412092	Myosin heavy chain. non-muscle isoform X4 (MYH9)	− 1.72	2.04E-02
A0A7M7LM34	LOC410058	Myosin light chain alkali isoform X1 (MYL1)	− 2.5	4.01E-03
A0A7M7IGQ8	552629	Superoxide dismutase (SOD)	2.84	2.75E-02
A0A7M7GSI8	LOC413867	Transaldolase (TALDO)	− 1.45	1.24E-02
B2D0J5		Venom carboxylesterase-6	2.57	3.73E-03

Using the UniProt and Panther tools, functional enrichment of the detected proteins revealed that they were differentially localized to the plasma membrane, extracellular matrix, mitochondria, cytoskeleton, and together, the myosin complex is associated with the treatment of both pesticides (Tables S4 and S5, respectively). Regarding molecular function, proteins associated with binding to biomolecules such as ATP, lipids, microtubules, calcium, heme group, and proteins associated with carbohydrate metabolism and the transport of proteins were found. Biological processes associated with differentially expressed proteins included carbohydrate metabolism, cytoskeletal reorganization, proton transport at the mitochondrial level, lipid transport and response to bacterial agents, and oxidative stress.

### Protein networks and physiologic processes affected in the thorax–abdomen fractions of *A. mellifera* treated with GLY and IMI

The protein networks differentially expressed in the thorax–abdomen of bees treated with GLY presented 56 nodes and 423 interactions (Figure S2a), whereas those detected in bees treated with IMI showed 61 nodes and 421 interactions (Figure S2b). Most of the detected proteins were clustered in the protein network, suggesting that the manifold of processes were affected by the treatments.

Table S6 presents the clustering data for the interaction network of differentially expressed proteins in the abdomen-thorax of bees treated with GLY and IMI. This table covers three types of clusters, displaying high scores and low False discovery rate (FDR) indices for proteins differentially expressed proteins. Clustering analysis of the identified proteins revealed information on cellular processes associated with carbohydrate metabolism, regulation of cellular structure, locomotion, energy production, and cellular stress response, among other functions (Table 6). In the GLY treatment, most of the processes were linked to neuromuscular systems and their functions, cell development and migration, and cellular homeostasis, among others. In contrast, IMI treatment showed implications for the response to oxidative stress, cellular reorganization, embryogenesis, muscle contraction, and glucose metabolism in general.

**Table 6:** Biological processes related to the clusters obtained from the protein interaction network of proteins detected in the thorax–abdomen of honeybees treated with GLY and IMI

Glyphosate (GLY)	Imidacloprid (IMI)
Actin cytoskeleton organization Assembly of cellular compounds Cell differentiation Cell growth-cell migration Embryonic development Head evolution Salivary gland morphogenesis Protein localization Sensory perception of sound (thoracic setae) Muscle contraction Homeostasis Lipid biosynthesis Gastrulation Translation of Rab protein signals Binding to Cu and Zn Cell movement Nervous system development Myosin filament assembly Actin filament organization	Glucose metabolism Carbohydrate metabolism Gluconeogenesis Organophosphate compound metabolism Glucose homeostasis Fructose 1.6-bisphosphate metabolism Vacuolar fusion regulation Oxidative phosphorylation Mitochondrial respiration ATP synthesis Electron transport in mitochondria from ubiquinol to cytochrome C Embryogenesis Neuronal differentiation Mitotic process regulation Actin cytoskeleton reorganization Cellular morphogenesis Cell migration Locomotion Superoxide dismutase (SOD) activity Respiratory system development Cytoplasmic/intracellular transport Muscle contraction Stress response

## Discussion

Pesticides have a significant impact on pollinators worldwide, but little is known about proteome changes. In this study, acute treatment with GLY or IMI was implemented in Africanized *A. mellifera* bees by administration through proboscis, ensuring that each individual received a defined homogeneous concentration and was observed for 24 h. Some studies describing the effects of herbicides such as GLY or insecticides such as IMI on honeybees often employ chronic treatments, with the herbicide administered topically or by ad libitum ingestion (Slater et al. 2020; Alburaki et al. 2022). These studies could be biased since honeybees have pesticide mixed with their food throughout the experiment, which could lead to the honeybee consumption of more or less than the desired concentration, generating heterogeneity in the results.

In this study, proteomics was performed in the head and thorax–abdomen fractions of honeybees treated acutely for 24 h with GLY at a sublethal concentration of 20 µg/bee and with IMI at a concentration of 2.4 ng/bee. In terms of behavior, no significant changes were observed and there was no lethality caused by the pesticide treatment.

### GLY and IMI could affect behavior at the individual and population levels

Among the proteins detected with low expression compared to the control, some are associated with calcium binding. Both pesticides downregulated regulatory unit B of phosphorylase kinase, an enzyme with calcium (Ca^2+^) binding capacity, in honeybees. Calcium plays a crucial role not only in muscle contraction but also in different signaling pathways and neurotransmitter syntheses (Rodwell 2015; Kadala et al. 2019). Deficiency of this enzyme could affect the sensory processes, learning, short-term memory (Farina et al. 2019), and loss of orientation and ability to obtain the food of honeybees exposed to these pesticides (Déglise et al. 2002; Lin et al. 2023a, b).

Another protein that showed reduced expression compared to the control in honeybees treated with IMI was the protein associated with the endoplasmic reticulum membrane complex (EMC4). This protein is involved in multiple processes at the neural tissue level in honeybees, including neural processes associated with the local or global presence of Ca^2+^. This protein is a membrane protein that participates in brain processes in honeybees of different ages because neural processes are age-dependent, including phosphorylation, endoplasmic reticulum protein processing, phagosome formation, endocytosis, and some signaling pathways related to protein synthesis and degradation, energy production, and signal transduction for brain function (Han et al. 2017). Additionally, the calcineurin B (CHP1), a protein known as calcium/calmodulin-dependent phosphatase that participates in neuronal plasticity, calcium homeostasis and is part of the immune system in mammals, was downregulated (Bueno et al. 2002; Kang et al. 2007; Saraf et al. 2018). In the *Drosophila melanogaster* model, this protein has been associated with the development of thoracic muscles (necessary for flight), regulation of circadian rhythm, axonal transport, mitochondrial function, associative olfactory learning, long-term memory and immune response to bacterial attacks (Shaw and Riederer 2003; Chang et al. 2003; Chang and Min 2005; Gajewski et al. 2006; Li and Dijkers 2015; Kweon et al. 2018; Wei et al. 2019). Thus, the deregulation of these proteins associated with IMI treatment may cause failures related to neural processes (Tan et al. 2015). This failure could be related to the detection system through olfactory associative learning and the chemoreceptor mechanisms located in the antennae of insects that allow the recognition of odors and pheromones could be simultaneously affected by showing low expression of the isoform of the neuronal sensory membrane protein.

The protein related to the circadian rhythm, period circadian protein (PER), which is expressed at lower levels than in the control, is responsible for regulating processes such as coordination, navigation, social behavior, time perception, communication through movements, and division of labor (Rubin et al. 2006). The low expression associated with GLY could impact the behavior of honeybees, as honeybees treated with sublethal concentrations of GLY showed slower navigation and a more complicated return path in maze trials (Zgurzynski and Lushington 2019).

Dysregulation of the balance of fatty acids in worker honeybees has been shown to cause variations in the ability to recognize odors, which affects flower selection and, consequently, pollination services (Bennett et al. 2022). The fatty acids omega-6 and omega-3 obtained through the diet are the most involved in learning processes (Arien et al. 2015, 2018). However, the presence of GLY may be associated with a deficiency of acetyl-CoA carboxylase (ACC), mevalonate kinase (MVK), and fatty acid synthase (FASN); thus, the total synthesis of fatty acids may not be efficient, affecting processes related to odor recognition and learning (Farina et al. 2019). This imbalance in odor recognition is further enhanced by the deficiency in the expression of Odorant Binding Protein 21 (OBP21), which is a protein detected in low expression and is related to aroma recognition mechanisms and is recognized by its respective receptors (Spinelli et al. 2012).

### GLY and IMI could affect metabolic processes in the head of *A. mellifera*

Carbohydrate metabolism can be affected when honeybees are treated with GLY. The expression of alpha-glucosidase (GAA) and phosphorylase b kinase (PHKB), which are related to carbohydrate metabolism, in the head was lower than that in the control. GAA is an enzyme present in the hypopharyngeal glands of honeybees (Kubota et al. 2004) and its function is to transform sucrose into glucose and fructose (Simpson et al. 1968; Kunieda et al. 2006). The reduction in the expression of this enzyme may be related to morphological variations generated by GLY in the hypopharyngeal glands, affecting energy production (Faita et al. 2018). Additionally, PHKB was found in high concentrations in the thorax–abdomen and even in the head of *A. mellifera carnica Pollm* (Panzenböck and Crailsheim 1997). This protein activates the enzyme glycogen phosphorylase to activate glycogenolysis for the release of glucose-1-phosphate, which is then be used for energy production (Rodwell 2015). However, the deficiency of this enzyme could generate a metabolic deficit associated with nutritional imbalances and protein synthesis associated with GLY (Faita et al. 2022). On the other hand, energy efficiency can be affected not only by the deficiency of these proteins but also by other factors related to the interaction network described in Table 6, in line with previous reports in honeybees treated with GLY associated with a mitochondrial protein deficiency (Cullen et al. 2023). Similarly, another report revealed that Roundup, a commercial GLY product, induces the uncoupling of oxidative phosphorylation in rat liver cells (Peixoto 2005). Thus, a reduction in protein levels or a failure of mitochondrial function can cause imbalances in metabolic processes related to energy deficiency.

Regarding fatty acid metabolism, it was found that GLY caused a reduction of enzymes such as acetyl-CoA carboxylase (ACC), mevalonate kinase (MVK), fatty acid synthase (FASN), and phosphomevalonate kinase (PMVK). All these enzymes are located in the cytosol, as shown in Table S1, indicating that the cytosol is a cellular component with a high percentage among the differentially expressed proteins. Each of these enzymes participates in different stages of fatty acid synthesis, the first being responsible for carboxylating acetyl-CoA to produce mevalonate and thus continuing the synthesis of fatty acids (Rodwell 2015). A reduction in the expression of these enzymes could affect processes associated with the synthesis of fatty acids for cell and tissue membranes, energy reserves, and the inhibition of bacterial growth associated with the presence of mandibular fatty acids (ten-carbon fatty acids), indirectly affecting the nutritional and pathogen response processes (Branchiccela et al. 2019). A reduction in bacterial growth inhibition is associated not only with a deficiency of fatty acids but also with the low expression of the major royal jelly protein 1 (MRJP-1), which has a nutritional, antimicrobial, immunogenic regulator, growth regulator functions, etc. (Mureşan et al. 2022). The reduction in MRJP-1 expression in honeybees treated with GLY could be associated with morphological changes in the hypopharyngeal and mandibular glands (Faita et al. 2018).

The MK enzyme is related to sterol synthesis; however, honeybees and insects, in general, lack the ability to synthesize sterols, including cholesterol. Instead, they require them through the diet by consuming phytosterols in this process (Jing and Behmer 2020). Among the functions of this enzyme are the synthesis of isoprenoid structures for the formation of juvenile hormones required in embryonic development processes, the repression of metamorphosis, the production of pheromones, and the synthesis of vitellogenin, among other functions (Bellés et al. 2005). Vitellogenin is an enzyme associated with resistance to oxidative stress, queen longevity, and the specialization of forager honeybees (Amdam and Omholt 2003; Nelson et al. 2007). Thus, MK deficiency could affect the processes described, and it has been shown that treatment of honeybees with sublethal doses of GLY can reduce the expression of vitellogenin affecting its related functions and even the response to oxidative stress (Castelli et al. 2021).

IMI generated a deficiency in the PHKB enzyme, suggesting that processes associated with energy production (glycogenolysis) may be affected, possibly due to mitochondrial dysfunction. This suggests that IMI affects the ability to metabolize substrates in the presence of oxygen and ATP synthesis (Nicodemo et al. 2014). Additionally, the deficiency of proteins such as apolipophorins (APO), which is responsible for lipid transport in the hemolymph could be associated with an energy imbalance in processes such as beta-oxidation caused by exposure to IMI (Parra-Peralbo and Culi 2011). Among the possible metabolic disorders associated with energy production, a downregulation of the isoform protein of alpha-tocopherol transferase (TTPA), which interacts with small molecules such as retinol, inositol, and vitamin E, was also found in the IMI treatment (Smith and Briscoe 2015). All of these molecules are required in the construction of cell membranes, so their deficiency could generate structural imbalances. Considering that it was detected at lower levels than in the control, it is possible to suggest that its deficiency could be associated with imbalances in oxidative stress events and could even affect cognitive processes associated with the presence of vitamin E, according to reports from studies in rat animal models (Joshi and Praticò 2012; Kiasalari et al. 2016). Like other vitamins, vitamin E is an essential nutrient for honeybees and is consumed (like other vitamins) through the ingestion of pollen; it is required for the feeding of adult honeybees, where nurses mostly consume this vitamin source (Elsayeh et al. 2022).

### IMI could impair intra- and extracellular proteins in the head

The detected cuticle protein 18.7 (CPR 18.7) belongs to the proteins that make up the insect cuticle (exoskeleton), part of the wings, and also the thoracic integumentary cuticle (Masson et al. 2018; Falcon et al. 2019). This protein showed low expression compared to the control, and its importance lies in being a structural component and defense against insecticides in honeybees (Falcon et al. 2019). Therefore, its deficiency could increase susceptibility to bacterial agents because certain cuticular proteins exhibit an expression pattern associated with bacterial attacks in *A. mellifera* (Kim et al. 2022).

The filamin A protein (FLNA), a member of the filamin-type protein family, its expression was downregulated compared to the control. This family of proteins is found in vertebrates and invertebrates (including insects), and its function is associated with interactions between the cytoskeleton and the extracellular matrix. They are involved in multiple cellular signaling pathways and interact with receptors, ion channels, transcription factors, and adhesion proteins (Razinia et al. 2012). Therefore, the low expression of this protein related to exposure to IMI could affect neuronal processes, leading to alterations in learning, memory, and other associated cognitive processes. A previous study demonstrated that adult honeybees of the *Apis cerana* species treated with sublethal doses of 0.59 ng/bee (40 times less than the concentration applied in this study) showed a reduction in the acquisition processes of olfactory memory, specifically in short-term memory, as well as recovery of their sense of smell after 1 h of treatment (Tan et al. 2015). However, it is not possible to determine whether the deregulation of this protein is associated with neuronal damage involving memory processes. Nevertheless, it is interesting to consider the possibility that this insecticide can generate morphological damage at the neuronal level, since in other study models, it has been found that the exposure of adolescent and adult rats to this insecticide generates histological patterns of neuronal degradation (Abd-Elhakim et al. 2018).

### IMI could be related to alterations in the transcriptional response in the head

The expression of RNA pol II (DNA-directed RNA polymerase II subunit RPB11) decreased compared to that of the control in the head fractions of honeybees treated with IMI (Table 2). This protein is related to mRNA synthesis and, therefore, transcriptional response (Danforth et al. 2006). Thus, its deficiency could have consequences on cellular processes associated with protein synthesis. Consistently, it has been reported that the chronic and sublethal administration of IMI to worker honeybees results in a reduction in the expression of mRNAs encoding proteins associated with xenobiotic metabolism (P450), antioxidant mechanisms, and the immune response, among others (Chen et al. 2021).

### IMI could affect the expression of proteins related to stress in the head

Within the proteins in head fractions of honeybees treated with IMI whose expression was lower than that of the control group was heat shock protein 83 (HSP83). This protein is related to HSP90. This protein is expressed in all living organisms in response to stress such as temperature (high or low), infections, starvation, UV radiation, pesticides, and wounds (Zhang et al. 1998; Kanagasabai et al. 2011; Alqarni et al. 2019). It has been reported that the treatment of honeybees with IMI at low doses (0.025 ng/bee) results in the overexpression of HSP90 upon exposure to temperatures between 20 and 28 °C. However, when the dose was increased to 0.1 ng/bee, contradictorily, the expression of this protein decreased (Manzi et al. 2020). In this research, a concentration of 2.4 ng/bee was applied, which corresponds to a concentration almost 100 times greater than that implemented in other studies (such as that of Manzi and colleagues) that identified the overexpression of the mentioned protein. Additionally, in the same study by Manzi and colleagues, the authors mentioned that a low concentration of IMI could generate significant changes in the expression of HSP90, suggesting a dose-dependent response that activates or deactivates the expression of this type of protein. On the other hand, the protein isoform X1 alpha-crystallin B (CRYAB) is described by the PANTHER tool as an HSP-type protein, and similar to HSP90, it is possible that not being at the right levels, the bee not only has an inadequate response to different types of stress such as temperature changes or possible toxic agents but also possibly protein maturation processes will be affected by the chaperone activity offered by heat shock proteins, leading to the loss or degradation of protein activity (Miller and Fort 2018).

### IMI could reduce the response to xenobiotic agents in the head

Cytochrome-type enzymes are heme-thiolate molecules that participate in the synthesis and metabolism of multiple endogenous substrates (steroid hormones, lipophilic signaling molecules, etc.) and xenobiotic activation or detoxification processes (Werck-Reichhart and Feyereisen 2000). Cytochrome P450 is one of the most studied and cytochrome B5 (CYB5) (which was detected at low levels in this research) has the ability to mediate the catalytic activity of P450 (Guzov et al. 1996; Murataliev et al. 2008). Similarly, a study reported that the exposure of honeybees to sublethal doses of IMI causes an increase in the cytochrome P450 expression as a possible detoxification mechanism (De Smet et al. 2017). In contrast, another study reported that this enzyme has a reduced expression (Chen et al. 2021). However, in this study, cytochrome P450 was not detected, but CYB5, which, as mentioned earlier, participates as an activator of cytochrome P450. It is important to note that IMI can activate certain detoxification mechanisms, and they may not be as efficient when low expression of other molecules required in multiple detoxification processes occurs, as is the case with the one detected in this study (CYB5). In addition to this possible imbalance in detoxification mechanisms, there is the low expression of the protein UDP-glucuronosyltransferase 2C1 (UGT2C1), which is also involved in processes related to xenobiotic metabolism associated with oxidative stress (Cui et al. 2020) and its deficiency could enhance the low response of honeybees to neonicotinoids such as IMI, as demonstrated by Chen and colleagues who found that by treating nurse honeybees for 11 days with IMI (0.2 ng/bee), 130 genes associated with detoxification processes were deregulated (Chen et al. 2021).

### GLY could affect the organization of the cytoskeleton in the thorax–abdomen

Proteins related to the cytoskeleton whose expression was lower than that of the control were the non-muscle isoform of myosin heavy head protein and TRAF3-1 interaction protein (TRAF3IP1). These proteins are associated with binding to actin filaments and ATP, motor regulation of the cytoskeleton, and microtubule binding, respectively. Myosins are proteins that can be either muscular or non-muscular; the latter participate in the development of cellular structure, while muscle myosins in insects are involved in the development of the muscular system from larvae to adulthood (Tarver et al. 2012). The detected proteins are essential for various cellular development processes, such as cell division, cell migration, remodeling, and intracellular transport. The reduction in their expression could be linked to cellular dysregulation. A previous study on bee larvae chronically exposed to sublethal doses of GLY revealed reduced weight gain associated with physiological cellular alterations that affect nutrition (Vázquez et al. 2018). Aspects of cellular development may manifest differently across cellular processes and are model-dependent. For instance, in the sea urchin species *Sphaerechinus granularis*, GLY was found to disrupt the cell cycle, as evidenced by reduced cell cycle times (Marc et al. 2004). In another study using the fish *Poecilia reticulata*, effects of GLY were observed in liver tissue, including steatosis, increased distribution of collagen fibers, and elongated hepatocytes, among other alterations (Antunes et al. 2017).

Similar to what was previously described for the head of *A. mellifera*, a reduction in the expression of MRJP-1, was detected in the thorax–abdomen in association with nutritional processes and antimicrobial response (Faita et al. 2018). Additionally, a decrease in the expression of apolipophorin-III-like protein precursor (APOIII), a protein associated with extracellular lipid transport, was detected. The absence or reduction of this protein could cause nutritional imbalances not only mediated by mitochondrial damage but also by a deficiency in the transport of lipid biomolecules necessary for the acquisition of energy and the formation of membranes.

Interestingly, a protein with a serine/arginine repetitive motif was detected with low expression compared to the control. This protein is relatively unknown in *A. mellifera*. However, it is found in both vertebrates and invertebrates, and functions related to restoring splicing activity for specific differentiation transcripts at the neuronal level are attributed (Richardson et al. 2011). Chronic exposure of *Apis mellifera* larvae to sublethal doses of GLY induces transcriptional changes associated with nutritional imbalances related to lipid metabolism, among other physiological alterations (Vázquez et al. 2020).

### IMI could increase the response to oxidative stress in the thorax–abdomen

As part of the response generated by honeybees to IMI, overexpression of the carboxylesterase 6 enzyme, which is present in bee venom and is associated with responses to insects and cytotoxic agents such as organochlorine pesticides, carbamates, or pyrethroids, was observed (Hemingway et al. 2004). It has been reported that exposure of *Apis cerana* bees to sublethal doses of IMI results in low expression of this enzyme, although these values are not statistically significant (Gao et al. 2020). In contrast, a study in which Colorado potato beetles were treated with 0.079 ng/μL IMI for 24 h demonstrated an increase in this enzyme. The authors suggested that the upregulation could be part of a detoxification mechanism of the beetle against the insecticide and even against a fungicide (chlorothalonil) evaluated in separate experiments (Clements et al. 2018). In addition to this venom-associated enzyme, the enzyme superoxide dismutase (SOD) was also found to be overexpressed in the thorax–abdomen of honeybees treated with IMI compared to the control. SOD is part of the antioxidant system that balances reactive oxygen species (Farjan et al. 2012). A study on SOD expression levels revealed that topical exposure of honeybees to IMI at a concentration of 20 ng/μL caused SOD overexpression in worker honeybees but low SOD expression in queen honeybees (Chaimanee et al. 2016). However, another study reported that the exposure of honeybees to food mixed with IMI at 10 ng/μL resulted in reduced expression of this enzyme (Gregorc et al. 2018). This variability suggests that detoxification mechanisms are diverse and dependent on insecticide administration, as oral administration, as implemented in this study, may activate detoxification mechanisms differently than topical or food-based applications.

Similar to the detection of proteins in the thorax–abdomen of honeybees treated with GLY, those treated with IMI exhibited low expression of proteins related to the metabolism associated with energy production. Examples include ATP synthase, glyceraldehyde 3-phosphate dehydrogenase (GAPDH), MRJP, FASN, MVK, and cytochrome C (CYCS). The latter is crucial for oxidative phosphorylation in the mitochondria to produce ATP from the dehydrogenation of the coenzymes NADH and FADH_2_ (Rodwell 2015). These results suggest that several processes related to energy production and other metabolic events may be affected. This finding is consistent with the findings of Paleolog and colleagues who reported that honeybees of the species *Apis mellifera carnica* exposed to sublethal doses of IMI may experience deficiencies in metabolic processes associated with nutritional disorders. This study revealed that honeybees treated with IMI exhibited a 69% deficiency of bioelements, including Na, K, Ca, Mg, Fe, Mo, Co, Cu, Ni, Se, and Zn, compared to those in the control group (Paleolog et al. 2023).

### IMI could affect thorax–abdomen section coordination in honeybees

The myosin heavy chain protein, non-muscle isoform X4 (MYH9), was also detected at low levels in the thorax–abdomen of honeybees treated with GLY. This protein is a key structure for the organization of the cytoskeleton and also forms the muscle fibers present in insects (Vigoreaux et al. 1991). Therefore, the low expression of this protein in this study suggests that IMI could cause structural damage at the cellular level, affecting motor processes associated with muscle failure. This is supported by the findings of Wu and colleagues, who treated honeybees chronically with IMI and detected low expression of genes encoding proteins associated with motor processes, which was also confirmed with the climbing test, which showed that these honeybees exhibited variations in this test compared to control honeybees (Wu et al. 2017).

In summary, the proteins detected in the head and thorax–abdomen of honeybees treated with GLY and IMI at the mentioned concentrations could cause damage associated with metabolic imbalances of carbohydrates and fatty acids, as well as learning processes and cytoskeletal reorganization, thus affecting protein synthesis and the immune response to cytotoxic agents. Additionally, this study identified common proteins between the two tissues evaluated in each treatment (see Fig. 1).

Agricultural products such as GLY and IMI are formulated with adjuvant compounds or surfactants, which enhance the absorption and therefore the efficacy of the active compound (Lin et al. 2023a, b). In our study, we used pesticides with commercial formulations, making it difficult to assess whether the observed changes in protein expression are attributable to the action of the active compound, the surfactant, or a synergistic effect between the two components. Currently, there are no proteomic studies focused on physiological variations in honeybees treated with acute doses of pure GLY and IMI. However, there are studies in which the variation in the learning processes of bumblebees (insects also belonging to the *Apidae* family) treated with pure GLY or its commercial formulation was evaluated, revealing a reduction in cognitive processes caused by GLY and not by the components present in the commercial formulation (Kaakinen et al. 2024).

On the other hand, there are currently no proteomic studies focused on physiological variations in honeybees treated with pure IMI or commercial formulations. The commercial formulation used in our honeybee treatments (Confidor-Bayer™) contained not only the active compound but also glycerol and 1,2-benzisothiazol-3(2H)-one which were not expected to produce any side effects. In honeybees, glycerol participates in antioxidant synthesis processes, fatty acid metabolism, and cryoprotection (Prado et al. 2022); regarding 1,2-benzisothiazol-3(2H)-one, it has been described in bumblebees that this compound does not cause changes in sucrose consumption, gut melanisation, or a reduction in mortality rate (Straw and Brown 2021). According to the aforementioned, it is important to mention that the proteomic changes found, and the possible physiological associations are related to the active components present in the GLY and IMI formulations we used in this study.

## Conclusions

Acute treatment of Africanized *A. mellifera* with sublethal doses of GLY and IMI resulted in changes in protein expression, allowing the detection of 49 downregulated proteins. Most of the detected proteins exhibited low expression compared to the control. This could suggest that acute exposure could cause alterations in protein synthesis in response to the initial phases of adaptation to a cytotoxic agent. Among the differentially expressed proteins, two proteins were found to be upregulated in the thorax–abdomen of honeybees treated with IMI compared to those in the control group. This may suggest that these proteins are part of the initial detoxification mechanisms or response to cellular stress caused by this insecticide. It is necessary to extend this type of research, based on the approach given in this study, to the assessment of changes in the proteome with chronic treatments, in order to evaluate expression variations over time of exposure to pesticides.

### Supplementary Information

Below is the link to the electronic supplementary material.

Supplementary file1 (DOCX 667 KB)
