# Supplementary material for: Changes in the proteome of Apis mellifera acutely exposed to sublethal dosage of glyphosate and imidacloprid
Source: Environ Sci Pollut Res Int. 2024 Jul 9;31(33):45954–69. doi: 10.1007/s11356-024-34185-x (PMC11269427; doi:10.1007/s11356-024-34185-x)
Supplement: Supplementary file 1 — Supplementary file1 (DOCX 667 KB) [file 11356_2024_34185_MOESM1_ESM.docx]

**Changes in the proteome of *Apis mellifera* acutely exposed to sublethal dosage of glyphosate and imidacloprid**

Carlos Andrés Maya-Aguirre^1,2^, Angela Torres^3^, Luz Dary Gutiérrez-Castañeda^2^, Luz Mary Salazar^3^, Yael Abreu-Villaça^4^, Alex C. Manhães^4^, Nelson E. Arenas^5^*

^1^ Instituto de Biotecnología, Facultad de Ciencias, Universidad Nacional de Colombia, Ciudad Universitaria, Avenida Carrera 30 N° 45–03, Bogotá, D.C., Colombia.

^2^ Grupo Ciencias Básicas en Salud – CBS-FUCS, Fundación Universitaria de Ciencias de la Salud, Hospital Infanti­l Universitario de San José, Carrera 54 No.67A – 80, Bogotá, D.C., Colombia.

^3^ Departmento de Química, Facultad de Ciencias, Universidad Nacional de Colombia, Ciudad Universitaria, Avenida Carrera 30 N° 45–03, Bogotá, D.C., Colombia.

^4^ Laboratório de Neurofisiologia, Departamento de Ciências Fisiológicas, Instituto de Biologia Roberto Alcantara Gomes, Universidade do Estado do Rio de Janeiro (UERJ), Rio de Janeiro 20550-170, RJ, Brazil

^5^ Facultad de Medicina, Universidad de Cartagena, Campus Zaragocilla, Barrio Zaragocilla, Carrera 50a #24-63, Cartagena de Indias, Bolívar, Colombia.

* Corresponding author: [narenas@unicartagena.edu.co](mailto:narenas@unicartagena.edu.co)

**Figure S1** Protein interaction network of differentially expressed proteins in the heads of honeybees treated with GLY (a) and IMI (b).

| **a** | 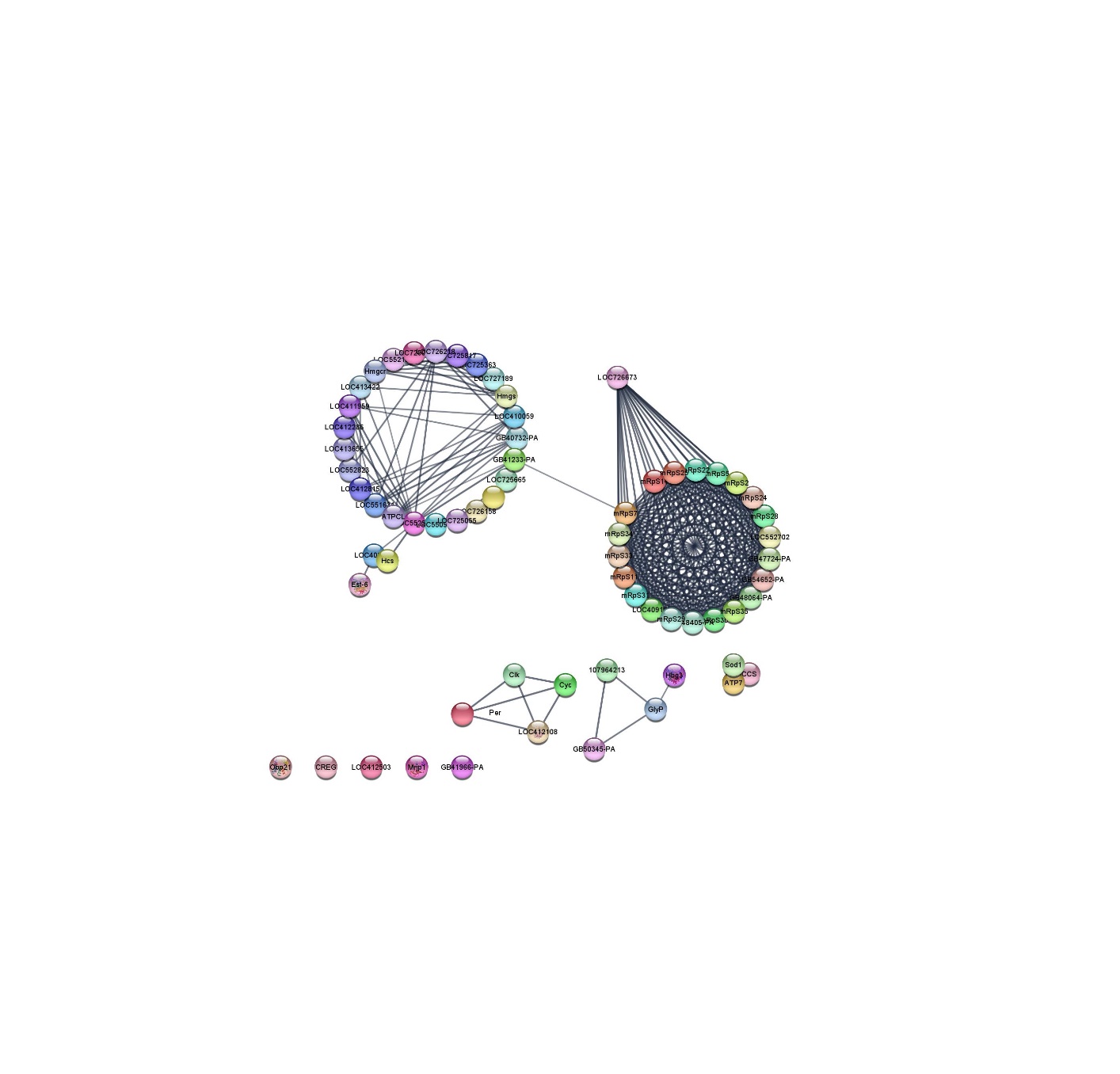 |
| --- | --- |
| **b** | **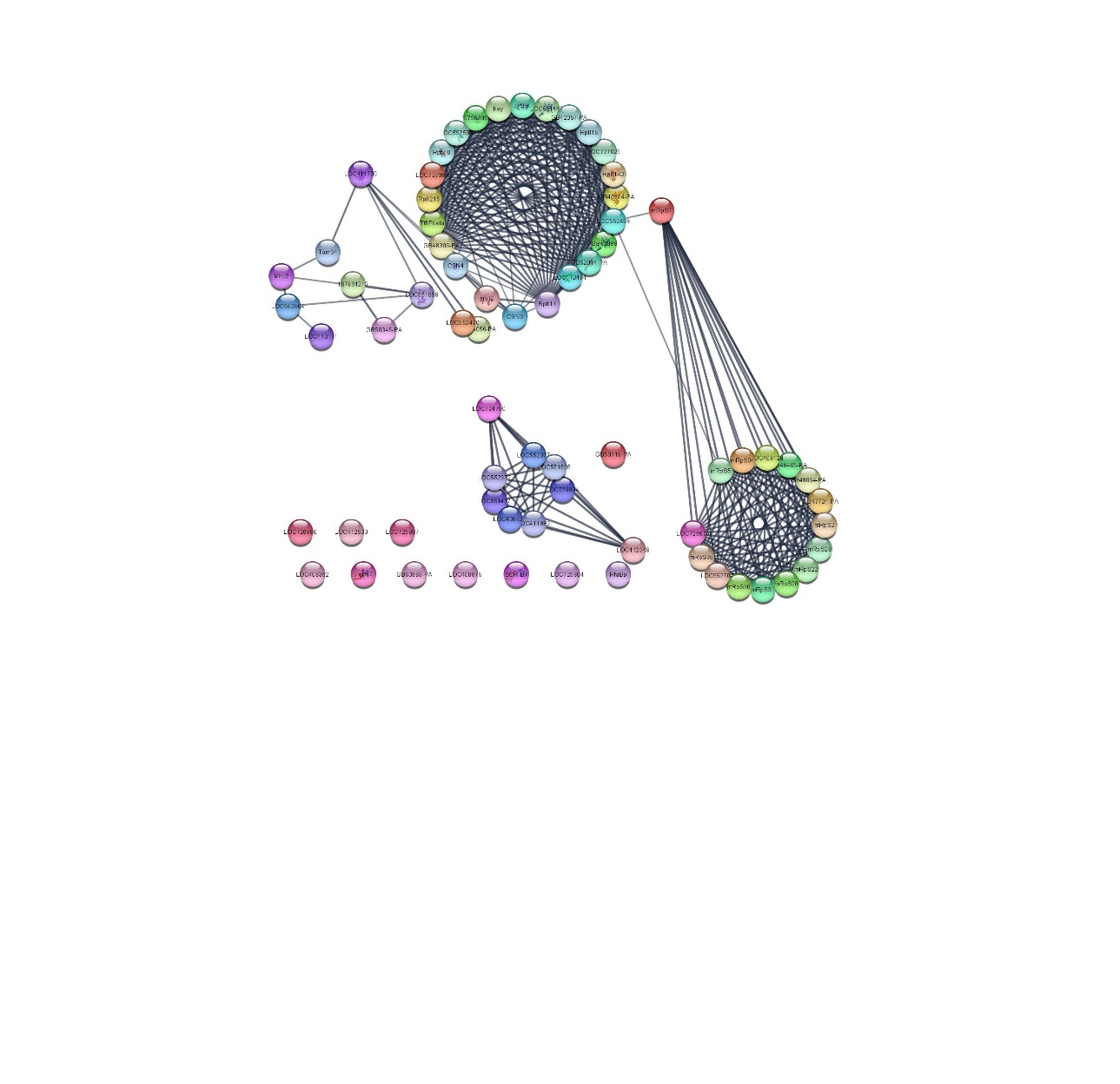** |

**Figure S2** Protein interaction network of differentially expressed proteins in the abdomen-thorax samples of honeybees treated with GLY (a) and IMI (b).

| **a** | **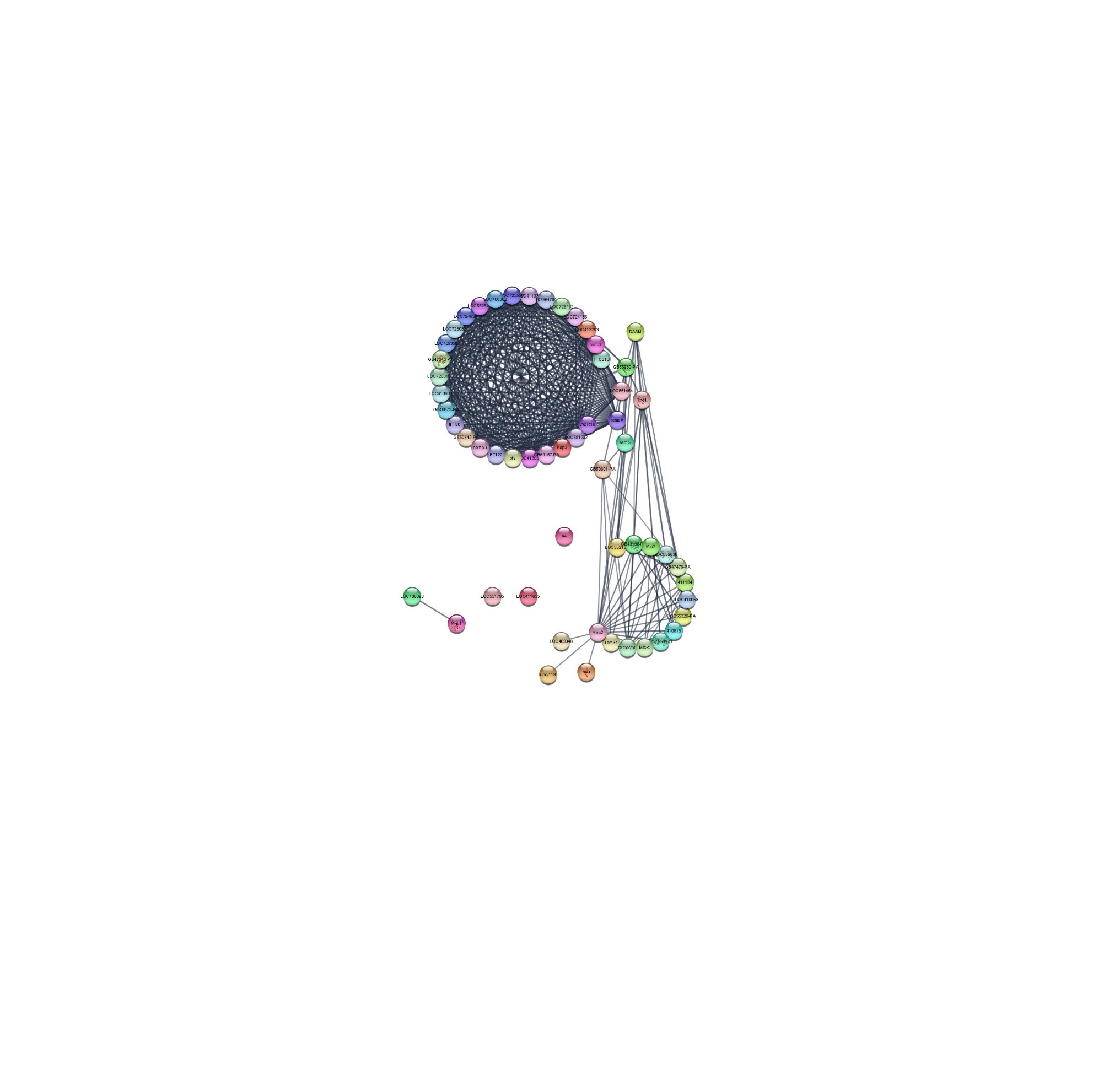** |
| --- | --- |
| **b** | **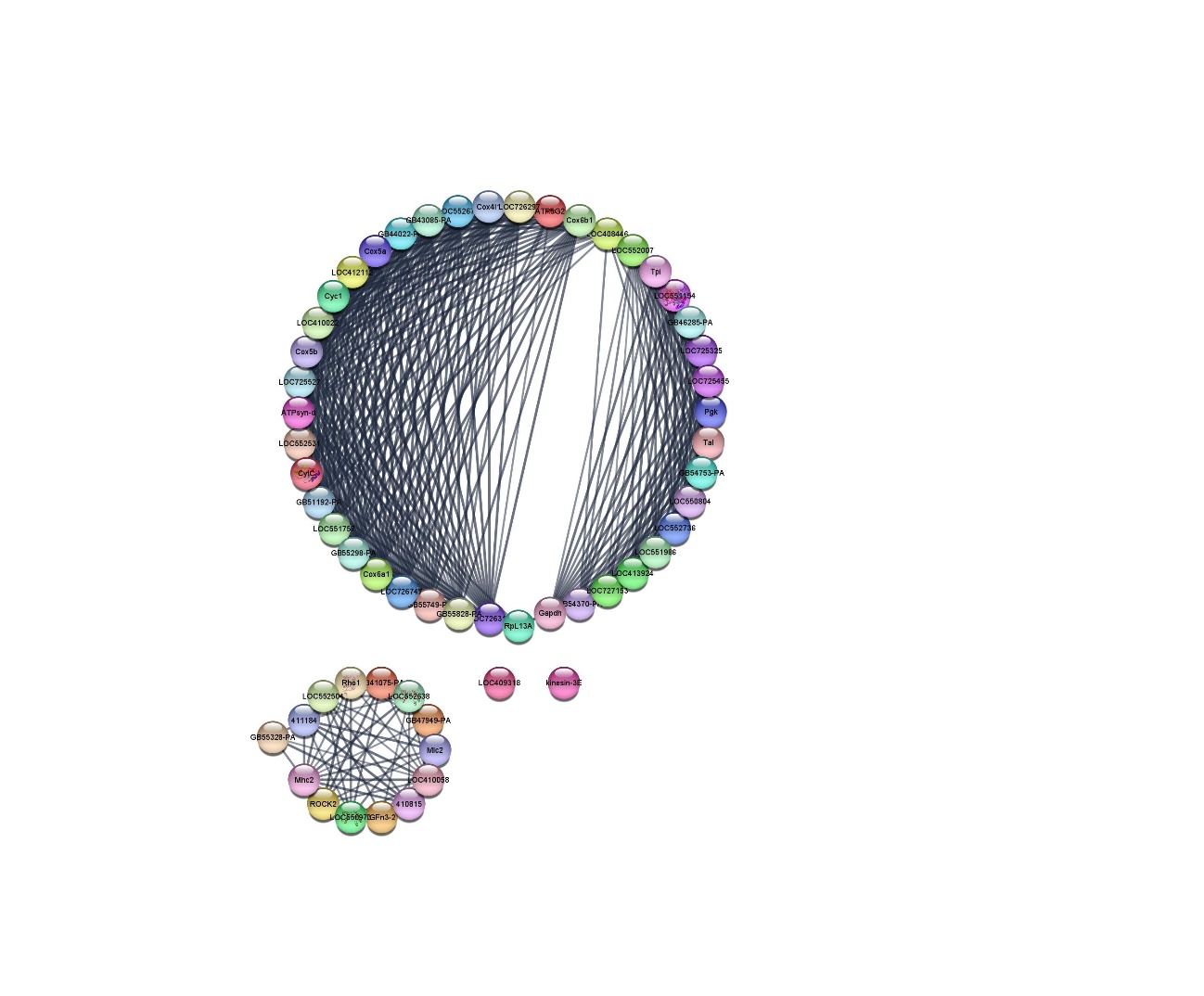** |

**Table S1** Functional enrichment of differentially expressed proteins in the head samples of honeybees treated with GLY compared to the control, classified according to the three annotations. Number of proteins (in parentheses)

| **Annotation** | **Description** |
| --- | --- |
| Cellular component | Extracellular Matrix (n=3), Cytosol (n=2), Nucleus (n=1), Plasma Membrane (n=2), Ribosomes (n=1), Mitochondria (n=2) |
| Molecular Function | Acetyl CoA Carboxylase (n=1), ATP Binding (n=2), Metal Ion Binding (n=1), Odorant Binding Protein (n=1), Calmodulin Binding (n=1), Transmembrane Metal Ion Transport Protein (n=1), Acyltransferase (Oxoacyl Synthetase) (n=1), Gamma-Glutamylcyclotransferase Activity (n=1), Carboxylesterase (n=1) |
| Biological process | Determination of Castes by Environmental Factors; Defense against Gram-negative and Gram-positive Fungi and Bacteria; Destruction of Cells from OtherOrganisms (n=1), Carbohydrate/Glycogen Metabolism (n=2), Fatty Acid Biosynthesis; Malonyl CoA Biosynthesis; Cholesterol Biosynthesis, Isopentenyl Pyrophosphate Biosynthesis; Mevalonate Pathway; Acyl CoA Metabolism, Phospholipid Biosynthesis; Phosphorylation (n=5), Development of Anatomical Structures; Regulation of Transcription by RNA Polymerase II (n=1), Mitochondrial Transmembrane Transport; Transmembrane Transport of Metal Ions; Biosynthesis of Secondary Metabolites (n=1) |

**Table S2** Functional enrichment of differentially expressed proteins in the heads of honeybees treated with IMI according to the three annotations compared to those in the control group. Number of proteins (in parentheses)

| **Annotation** | **Description** |
| --- | --- |
| Cellular component | Extracellular Matrix (n=1), Cytosol (n=2), Nucleus (n=1), Plasma Membrane (n=8), Ribosomes (n=1), Mitochondria (n=1), Endoplasmic Reticulum (n=1), Myosin Complex (n=1) |
| Molecular Function | Structural Molecule (n=1), Lipid Transport (n=1), Calcium Ion Binding (n=1), Heme Group Binding (n=1), DNA Binding (n=1), Transmembrane Metal Ion Transport Protein (n=1), Unfolded Protein Binding (n=1), UDP-Glycosyltransferase Activity (n=1), Calmodulin Binding (n=1), Actin Filament Binding (n=2) |
| Biological process | Cellular Response to Heat; Protein Folding and Stabilization (n=1), Mitochondrial Transmembrane Transport; Transmembrane Transport of Metal Ions; Biosynthesis of Secondary Metabolites; Fatty Acid Biosynthesis (n=1), Protein Folding (n=2), Glycogen Metabolism (n=1), Organization of Actin Cytoskeletal Structures (n=1) |

**Table S3** Clustering data for the interaction networks of differentially expressed proteins in the heads of honeybees treated with GLY and IMI. False Discovery Rate (FDR).

| **Network** | **Number of Clusters** | **Clusters** | **Nodes** | **Edges** | **Score** | **FDR** |
| --- | --- | --- | --- | --- | --- | --- |
| GLY | 6 | 1 | 22 | 231 | 22000 | 2,3X10^-47^ |
|  |  | 2 | 14 | 45 | 6923 | 7,4X10^-47^ |
|  |  | 3 | 4 | 6 | 4000 | 1,31X10^-42^ |
|  |  | 4 | 4 | 6 | 4000 | 5,34X10^-30^ |
|  |  | 5 | 3 | 3 | 3000 | 5,29X10^-15^ |
|  |  | 6 | 3 | 3 | 3000 | 0,0126 |
| IMI | 2 | 1 | 36 | 311 | 17771 | 1,61X10^-21^ |
|  |  | 2 | 9 | 36 | 9000 | 8,75X10^-15^ |

**Table S4** Functional enrichment of differentially expressed proteins compared to the control in the thorax-abdomen samples of honeybees treated with GLY. The number of proteins within parentheses.

| **Annotation** | **Description** |
| --- | --- |
| Cellular component | Extracellular Matrix (n=2), Cytosol (n=1), Plasma Membrane (n=2), Myosin Complex (n=1) |
| Molecular Function | Lipid Binding (n=1), ATP Binding (n=1), Microtubule Binding (n=1) |
| Biological process | Determination of Castes by Environmental Factors; Defense against Gram-negative and Gram-positive Fungi and Bacteria; Destruction of Cells from Other Organisms (n=1), Lipid Transport (n=1), Regulation of Cytoskeletal Microtubule Organization; Intraciliary Transport Involved in Cilium Assembly; Axoneme Assembly; Vesicular Trafficking from Golgi to Periciliary Membranous Compartments (n=1) |

**Table S5** Functional enrichment of differentially expressed proteins in thorax-abdomen samples from honeybees treated with IMI compared to those from control bees. The number of proteins within parentheses.

| **Annotation** | **Description** |
| --- | --- |
| Cellular component | Cytosol (n=1), Mitochondria (n=2), Myosin Complex (n=1) |
| Molecular Function | Transaldolase Activity (n=1), ATP Binding (n=2)Proton Transmembrane Transport Activity (n=1), Calcium Ion Binding (n=2), Heme Group Binding (n=1), Glyceraldehyde-3-Phosphate Dehydrogenase Activity (n=1), Superoxide Dismutase (SOD) Activity (n=1) |
| Biological process | Carbohydrate Metabolism; Pentose Phosphate Pathway Bypass (n=1), Microtubule-Based Movement (n=1), Proton Movement for ATP Synthesis (n=1), Glucose Metabolism (n=1), Superoxide Dismutase (SOD) (n=1) |

**Table S6** Clustering data for the interaction networks of differentially expressed proteins in the heads of honeybees treated with GLY and IMI. False Discovery Rate (FDR).

| **Network** | **Number of Clusters** | **Clusters** | **Nodes** | **Edges** | **Score** | **FDR** |
| --- | --- | --- | --- | --- | --- | --- |
| GLY | 3 | 1 | 27 | 322 | 24769 | 6,51X10^-51^ |
|  |  | 2 | 9 | 30 | 7500 | 3,29X10^-23^ |
|  |  | 3 | 5 | 9 | 4500 | 1,09X10^-09^ |
| IMI | 3 | 1 | 22 | 226 | 21526 | 8,58X10^-37^ |
|  |  | 2 | 16 | 113 | 15067 | 3,35X10^-27^ |
|  |  | 3 | 11 | 36 | 7200 | 6,91X10^-23^ |
